# A Case of Thrombosis of the Pulmonary Artery

**Published:** 1901-12

**Authors:** E. L. Quitman

**Affiliations:** Chicago, Ill.


### REPORTS OF CASES.

### A CASE OF THROMBOSIS OF THE PULMONARY ARTERY.

By E. L. Quitman, M.D.C.,
CHICAGO, ILL.

During the evening of September 27, 1901, I was called to the stable of one of my clients to see a mare about nine years old, weighing in the neighborhood of 1100 pounds, which was thought to have been "foundered."

The history of the case was that the mare had been idle for about four weeks, but had been at work for the past four or five days. Previous to this time she had never been sick or ailing during the time of her present ownership, which was over three years, and her idleness was due to the owner having no use for her.

She was noticed to be stiff in the morning, but I was not called until evening. On going into the stable the attendants were found trying to move her, and my first impression, gained from her attitude and apparent disinclination to move, was that I had a case of laminitis, myositis, or muscular rheumatism to deal with; but upon examining the pulse, which was my first step in the examination, I saw that I had something "unique" to deal with, and immediately put on my thinking-cap. I found a pulse which was at least one-half inch in diameter, extremely resistant, and beating like a trip-hammer. This in an exaggerated sense also suggested lymphangitis. The accompanying vein was also immensely distended, but more compressible.

I next examined all superficial bloodvessels, and found all extremely distended and firm. Especially noticeable were the larger bloodvessels on the inner aspect of the limbs, both hind and fore, which seemed at least an inch in diameter. The mucous membranes were slightly injected. Respiration rapid—about 50 to 60 per minute. Occasionally there would be muscular contraction of the trunk, as though affected with "shooting pains," lasting but a moment. Surface temperature was apparently normal, or higher than normal; temperature per rectum, 104.5° F.

The limbs were warm and lung-sounds normal. There was apparently an inability for motion, but upon forcing the animal this proved to be a disinclination rather than an inability. On first attempt the mare would settle back on her hindquarters, as if badly foundered; then, getting started, would act as though the front limbs were cramped or "asleep," and after getting headway always under force, would walk fairly well. Her countenance showed no signs of pain or distress except for a moment, when the "shooting pains" occurred. The bowels were normal, and the appetite excessive rather than lessened.

After carefully weighing all facts and excluding apparent ailments, particularly rheumatism, for want of completing symptoms, I made a diagnosis of thrombosis of some large internal vessel, even mentioning the pulmonary artery as the probable seat, on account of the increased respirations. I felt reasonably sure that it was not in the femoral or the axillary arteries (which is the most common seat), on account of all four limbs showing trouble, with possibly more marked manifestation of the ailment anteriorly; but there was no coldness, and the symptoms did not abate after rest. The quickened and increased respiration suggested the pulmonary artery, or at least the anterior aorta, though this would occur, but to a less extent, in thrombosis of the posterior aorta.

The prognosis was unfavorable. I put the animal under treatment consisting mainly of iodide of potash and carbonate of ammonia, alternated. The temperature varied from 103° F. to 105° F. during the progress of the ailment. No organic or inflammatory disease was manifested until the last four or five days of the animal's existence; then gangrene of the lungs made its appearance, without any preliminary symptoms of pneumonia. The animal never coughed, even after the twenty-sixth or twenty-seventh day, when I detected a most fetid odor of her breath. I then depressed her head, which would cause a profuse flow of a very fetid, seropurulent, slightly bloody fluid from her nostrils. I then informed the attendant that gangrene of the lungs had set in, and that her end was not far off. Emaciation progressed from the start, though she ate full rations, even finishing a full feed of oats the evening preceding her death, which occurred during the night of October 20, 1901, twenty-three days after the commencement of her ailment.

I should add that on about the tenth day she showed an improvement to such an extent that it seemed her recovery was assured; but after a couple of days of this apparent improvement she relapsed into her former condition, without material change until the pulmonary gangrene set in.

*Post-mortem.* I have never before seen so many pathological conditions in one animal. It was simply remarkable that the mare lived so long. In the abdominal cavity there was found some effusion, patchy peritonitis, bowels congested, kidneys enlarged, stomach congested, spleen empty, flat and flabby, and beautifully mottled with hemorrhagic infarcts. The ovaries were normal. The liver had an old cicatrix on it about one and a half by one inch in size, and a number of small elevations of Glisson's capsule, as if gas had forced it out into minute, elongated pouches.

Upon opening the thoracic cavity a considerable amount of very fetid gas escaped. There were also some serous effusion here, patchy pleurisy, and a pericarditis. The heart was enlarged, and contained ante-mortem but recent clots. The lungs were filled and distended with a very foul serosanguineous fluid, which ran out when the lungs were cut, and could be squeezed out like water from a sponge. There were large and innumerable areas of gangrene, and were emphysematous.

Upon opening the pulmonary artery I came upon my object of search. There we found a thrombus of a yellowish or grayish-white color, which I caught hold of and gently pulled upon, and was rewarded by drawing out a clot which showed the distribution of the bloodvessels most beautifully and minutely. I spread this out on white paper, and the clot shows the main trunk, which represented the pulmonary artery and the right and left branches, into both lungs, and further subdivisions, until it became as fine as the finest hair.

It seemed almost impossible that a clot could get so tough as to have strength enough to allow it to be drawn out of the most minute bloodvessels. This toughness proves its age, and in my opinion leaves no doubt as to the diagnosis made on my first visit.

In conclusion, I will say that I based my diagnosis almost entirely on the very great distention of the bloodvessels, together with a lack of completing symptoms of other apparent ailments; or, in other words, I diagnosed "by exclusion." I figured that nothing short of a mechanical obstruction could cause such a condition of the bloodvessels and pulse, alongside of which a typical laminitis pulse, which I believe is our largest pulse, becomes a small, wiry pulse in comparison.

---

THE Cleveland, Ohio, Veterinary Medical Association has been recently organized with the following officers for 1901–1902: Dr. E. P. Schaffter, President; Dr. E. H. Shepard, Vice-President; and Dr. A. E. Cunningham, Secretary-Treasurer. The Association has sixteen members.

